# Investigation of Hepatitis E Outbreak Among Refugees — Upper Nile, South Sudan, 2012–2013

**Published:** 2013-07-26

**Authors:** Kerry Thomson, Dvorzak José Luis, John Lagu, Richard Laku, Brendan Dineen, Marian Schilperoord, Martin Muita, Stella Gikunju, Lilian Waiboci, Barry Fields, Eyasu Teshale, Gemechu Gerbi, Susan Cookson, Tom Handzel, Miriam Shiferaw, Kevin Clarke

**Affiliations:** Médecins Sans Frontières; Republic of South Sudan Ministry of Health; United Nations High Commissioner for Refugees, South Sudan; United Nations High Commissioner for Refugees, Geneva, Switzerland; CDC-Kenya; Div of Viral Hepatitis, National Center for HIV/AIDS, Viral Hepatitis, STD, and TB Prevention; Div of Global Health Protection (proposed), Center for Global Health, CDC

During the week of July 2, 2012, the deaths of two pregnant women and one child were reported by household mortality surveillance in Jamam refugee camp, Maban County, Upper Nile State, South Sudan. All were reported to have yellow eyes before death. During July 27–August 3, 2012, three adult males with acute onset jaundice were admitted to the Médecins Sans Frontières (MSF) hospital in Jamam camp; two died within 4 days of admission. The Republic of South Sudan Ministry of Health, United Nations High Commissioner for Refugees (UNHCR), CDC, and humanitarian organizations responded through enhanced case surveillance, a serosurvey investigation, and targeted prevention efforts. As of January 27, 2013, a total of 5,080 acute jaundice syndrome (AJS) cases had been reported from all four Maban County refugee camps (Doro, Gendrassa, Jamam, and Yusuf Batil). Hepatitis E virus (HEV) infection was confirmed in a convenience sample of cases in each camp. A cross-sectional serosurvey conducted in Jamam camp in November 2012 indicated that 54.3% of the population was susceptible to HEV infection. Across all camps, an AJS case-fatality rate (CFR) of 10.4% was observed among pregnant women. The outbreak response has focused on improving safe drinking water availability, improving sanitation and hygiene, conducting active case finding, and optimizing clinical care, especially among pregnant women. Sustaining these improvements, along with strengthening community outreach, is needed to improve outbreak control. Further investigation of the potential role for the newly developed HEV vaccine in outbreak control also is needed (1).

Refugees began fleeing armed violence in Blue Nile State, Sudan, in late 2011, initially settling in Doro, the oldest camp. By July 2012, the Maban County refugee camp population surged to 110,000, coinciding with the onset of heavy rains and flooding. Flooding disproportionately affected large sections of Jamam camp, forcing refugee relocation to Gendrassa camp, 12 miles (20 kilometers) away. Yusuf Batil camp, 2 miles (3 kilometers) from Gendrassa, also was rapidly settled during the 2012 population displacement. An acute humanitarian emergency ensued, with crude mortality rates exceeding the emergency threshold of one per 10,000 per day in July and August; diarrheal disease was a leading cause of morbidity and mortality.

UNHCR and World Health Organization consider AJS to be a priority syndrome for communicable disease surveillance in humanitarian emergencies (2). The South Sudan Ministry of Health case definition for AJS is acute onset of jaundice and severe illness in any person. The etiologies and outcomes of AJS are varied and represent multiple diseases of outbreak potential, including HEV. HEV is endemic in Sudan and South Sudan; however, the extent of immunity is unknown. Transmission is fecal-oral, with an incubation period of 2–8 weeks. Globally, the overall CFR for HEV has been reported to range from 0.2% to 4%; mortality in pregnant women can be as high as 10%–25% (3). No unique clinical manifestations of hepatitis E distinguish it from other viral AJS etiologies, such as hepatitis A or yellow fever.

Following the initial cluster of AJS cases in July 2012, active surveillance was implemented in each camp by training community health workers on detection and referral of jaundiced patients to MSF health facilities. Clinician-confirmed AJS cases were documented using standardized line lists. No diagnostic testing for HEV was available at the field level. The etiologic cause of the outbreak was confirmed as HEV in August 2012 by the CDC-Kenya Medical Research Institute laboratory in Nairobi, Kenya, after six of eight initial AJS cases from Jamam camp were positive by reverse transcription–polymerase chain reaction (rt-PCR) for HEV. Blood specimens were tested for alternative acute infectious hepatitis etiologies, specifically yellow fever and viral hemorrhagic fevers. All eight were negative for these alternative etiologies. Subsequent AJS cases from the three other camps also were confirmed as HEV positive by rt-PCR. After alternate etiologies were excluded and HEV was confirmed in each camp (38 of 62 [61.3%] AJS cases tested were rt-PCR positive for HEV), cases of AJS recognized clinically were considered probable cases of HEV. Dipstick testing for bilirubinuria was used as a diagnostic adjunct when a finding of yellow eyes was in doubt.

As of January 27, 2013, a total of 5,080 AJS cases were reported: 3,291 in Yusuf Batil, 1,261 in Jamam, 474 in Gendrassa, and 54 in Doro (Figure 1). During the first weeks of 2013, a large increase in cases was reported from Yusuf Batil, with a second peak observed in Jamam and Gendrassa. The initial peak had occurred in August 2012. Possible explanations for this second peak include: 1) less than optimal water, sanitation, and hygiene interventions, both at the community and household levels; 2) the long incubation period of HEV, resulting in an increase of cases well after control measures had been put in place; and 3) alternative modes of transmission, including person-to-person transmission. Median patient age was 25 years, and 52.5% were female. Among pregnant women, 211 AJS cases and 22 deaths were reported (CFR = 10.4%). UNHCR population estimates were used to calculate age-specific attack rates and risk ratios for AJS death among pregnant women. Approximately 2,027 women (3% of the total Jamam, Yusuf Batil, and Gendrassa population) were estimated to be pregnant, based on an assumed crude birth rate of 39 per 1,000 (4). The risk for death from AJS among pregnant women was estimated to be 4.8 times that for nonpregnant women aged 18–59 years in the three most affected camps (Jamam, Gendrassa, and Yusuf Batil). The overall attack rate in the three most affected camps was 7.4%; persons aged 18–59 years had the highest attack rates (Figure 2). As of January 27, 2013, a total of 576 (11.3%) AJS patients identified by surveillance had been hospitalized in the three most affected camps, with a cumulative hospital CFR of 17.5% (Figure 3). Of the 101 hospitalized patients who died, 51.5% were female; the median age was 29 years. Hospital data for Doro patients were limited.

The surge of AJS patients required a sustained medical response in challenging field conditions. MSF’s clinical response focused on supportive management. In addition to individual symptom management, all outpatients received multivitamins, supplemental nutrition, soap, and hygiene education. A concerted effort to improve community outreach was implemented. Outpatients were reassessed every 7 days until symptoms resolved. Patients with severe fever, anorexia, vomiting, diarrhea, bleeding, agitation, or coma were admitted, as were patients with a positive malaria rapid diagnostic test, hypoglycemia, or pregnancy. A low-threshold approach to hospitalization was taken, including admission of all jaundiced pregnant women for observation, because of challenges in predicting clinical course.

Critically ill patients had confusion, agitation, coma, hypoglycemia, or suspected electrolyte imbalances. These patients required intensive care in a resource-limited setting to manage fluid balance and complications of hepatic encephalopathy. Initial treatment included antibiotics and intravenous fluids. Metronidazole was administered if the mental status changed, and ceftriaxone was administered if fever or suspected bacterial infection was present. Intravenous dextrose and saline fluid were alternated to prevent hypoglycemia and hyponatremia, respectively, when enteral feeding was not feasible. Adjunctive haloperidol for agitation and vitamin K for coagulopathy were provided.

A cross-sectional serosurvey was conducted in Jamam camp during November 6–10, 2012, to estimate population susceptibility and understand potential outbreak evolution. A total of 443 randomly selected persons aged ≥3 years from households sampled by simple and systematic random sampling provided consent for anti-HEV antibody testing. The CDC-Kenya Medical Research Institute laboratory used enzyme immunosorbent assay kits to detect anti-HEV immunoglobulin M (IgM) and anti-HEV immunoglobulin G (IgG) among participants. Serology results were weighted for age, based on UNHCR population data, to be representative of the Jamam population at the time of the survey. Overall, 21.7% (CI = 17.6–25.7) had IgM anti-HEV, representing recent exposure to HEV, and 54.3% (CI = 49.2–59.3) had no serologic evidence of recent or prior HEV infection (i.e., both IgM and IgG negative).

## Editorial Note

Globally, an estimated 20 million HEV infections occur annually, resulting in 3.4 million cases of acute hepatitis and 70,000 deaths (5). HEV is the most common cause of acute viral hepatitis globally. Recent large outbreaks have occurred among displaced persons in Sudan, Chad, and Uganda (6). The first such outbreak documented in Africa occurred among Angolan refugees in Namibia in 1983 (7). The current outbreak in South Sudan shares similar epidemiologic characteristics with other HEV outbreaks. Similar to a 2007 outbreak in northern Uganda, this outbreak started during the rainy season and has had high attack rates among young adults and high mortality among pregnant women (8). The serosurvey conducted during this outbreak showed that more than half of Jamam camp residents had no evidence of recent or past HEV infection, suggesting that these persons remained uninfected and were still susceptible to HEV infection 3 months after the implementation of control measures.

Since the South Sudan Ministry of Health declared the HEV outbreak in September 2012, efforts to improve water, sanitation, and hygiene conditions have been ongoing. Health and hygiene promoters have been trained on HEV prevention and active case finding. HEV preventive hygiene education has been conducted during household visits, at health facilities, and in community forums. UNHCR and partner agencies have scaled up water, sanitation, and hygiene activities, including increasing the availability of treated drinking water, increasing latrine coverage, distributing soap and water storage vessels, installing handwashing stations, and expanding hygiene promotion activities. Further water, sanitation, and hygiene improvements are needed to address ongoing transmission.

Implementing outbreak control measures in displaced persons camps often is extremely challenging. Scaling up water, sanitation, and hygiene interventions takes time. In addition, the long HEV incubation period often complicates response because transmission can occur for weeks before symptomatic cases first appear. This was witnessed in the current Maban County outbreak, where despite an apparent decrease in HEV cases following the scaling up of water, sanitation, and hygiene measures, a second peak was observed several months later. A cross-sectional survey conducted in Jamam camp in November 2012 revealed that 54.3% of the Jamam population was susceptible to HEV, after the initial peak already had occurred. The high proportion of nonimmune persons, in conjunction with potential alternative routes of transmission (e.g., person-to-person), might explain the prolonged nature of this outbreak. Crowded living conditions also might facilitate multiple transmission routes, resulting in the need for improved hygienic conditions at the community and household levels (9). Unlike single-source waterborne outbreaks, HEV outbreaks in such settings can display a prolonged multimodal course (i.e., multiple peaks, each attributed to separate modes of transmission) and might not abate rapidly with targeted water, sanitation, and hygiene interventions. However, such interventions remain the main strategy to interrupt transmission. Given the difficulty in controlling HEV outbreaks in emergency settings, additional interventions, such as vaccination, need further consideration.

A recombinant, 3-dose series HEV vaccine is available but has not yet been prequalified by the World Health Organization. The vaccine has been shown to prevent symptomatic HEV infection and proven to be safe and effective in persons aged 16–64 years (1). Limited vaccine safety data in 37 pregnant women receiving 57 doses has been reported (10); however, further research is needed, and safety for children is unknown. The vaccine is expected to be protective against HEV genotype 1, the strain associated with most waterborne outbreaks in Africa and Asia. Several questions regarding duration of immunity and prevention of subclinical infection remain. The effectiveness and implementation logistics of a 3-dose vaccine in an outbreak setting, particularly a challenging setting such as a displaced persons camp, also needs investigation. Genotype testing on serum samples collected for the cross-sectional serosurvey has not been performed to date.

Large HEV outbreaks have occurred among crowded displaced populations. These outbreaks result in appreciable morbidity and mortality, particularly among pregnant women. Despite enhancing water, sanitation, and hygiene control measures, outbreaks often are prolonged and necessitate a sustained prevention and control response. The role of vaccination in the context of outbreak control urgently needs to be examined.

What is already known on this topic?

Hepatitis E virus (HEV), which is transmitted via the fecal-oral route, is the most common cause of acute viral hepatitis globally. Large HEV outbreaks have been documented in crowded settings that have poor water, sanitation, and hygiene conditions. Pregnant women suffer disproportionately high mortality from hepatitis E.

What is added by this report?

A hepatitis E outbreak in South Sudan has demonstrated ongoing transmission, possibly including person-to-person transmission, among refugees in crowded living conditions with poor water, sanitation, and hygiene conditions. Following the initial peak, 54.3% of the Jamam camp population remained susceptible to HEV infection, despite having traveled from a region where HEV is believed to be endemic. The outbreak has strained existing local and humanitarian relief health facilities, and additional resources are needed.

What are the implications for public health practice?

Given that HEV transmission has continued among refugees in South Sudan despite improvements in water, sanitation, and hygiene conditions, further consideration should be given to alternative methods of HEV outbreak control and response efforts. This outbreak also underscores the need for investigation of a possible role for an HEV vaccine.

## Figures and Tables

**FIGURE 1:**
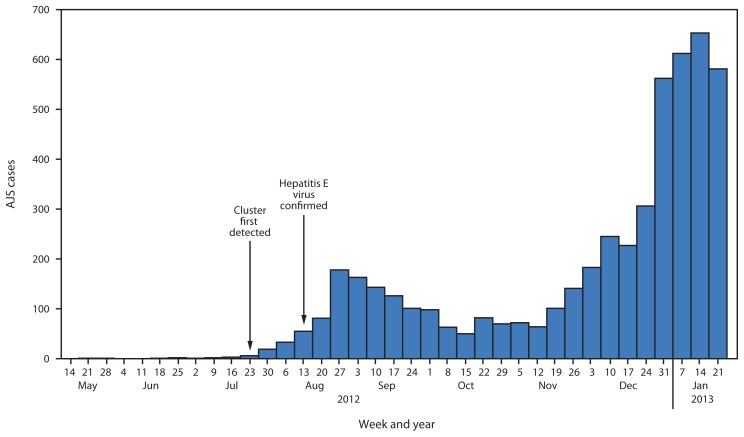
Acute jaundice syndrome (AJS) cases, by surveillance week — Jamam, Gendrassa, and Yusuf Batil refugee camps, South Sudan, 2012–2013

**FIGURE 2:**
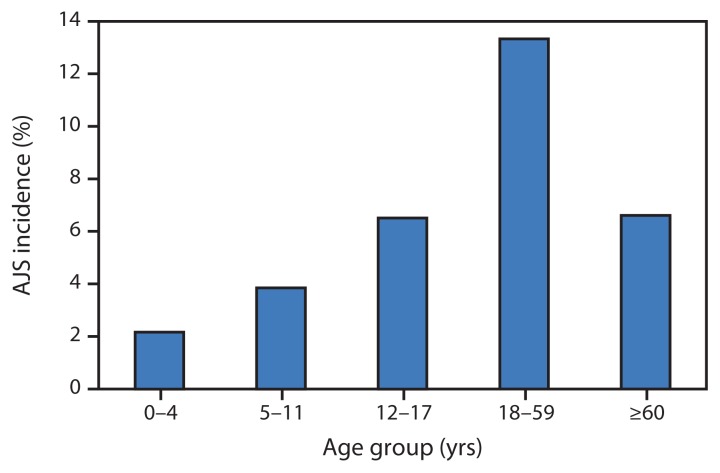
Cumulative acute jaundice syndrome (AJS) incidence, by age group — Jamam, Gendrassa, and Yusuf Batil refugee camps, South Sudan, July 2012–January 2013

**FIGURE 3:**
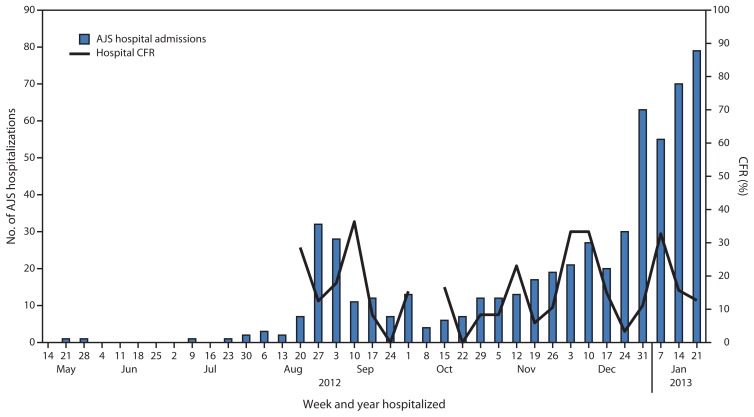
Acute jaundice syndrome (AJS) hospital admissions and weekly hospital case-fatality rate (CFR), by week of hospitalization — Jamam, Gendrassa, and Yusuf Batil refugee camps, South Sudan, 2012–2013* * Hospital CFR not reported if the number of AJS hospital admissions in a given week was ≤5.
